# Laboratory Evaluation of Natural and Synthetic Aromatic Compounds as Potential Attractants for Male Mediterranean fruit Fly, *Ceratitis capitata*†

**DOI:** 10.3390/molecules24132409

**Published:** 2019-06-29

**Authors:** Nurhayat Tabanca, Marco Masi, Nancy D. Epsky, Paola Nocera, Alessio Cimmino, Paul E. Kendra, Jerome Niogret, Antonio Evidente

**Affiliations:** 1 United States Department of Agriculture, Agricultural Research Service, Subtropical Horticulture Research Station (SHRS), Miami, FL 33158, USA; 2 Department of Chemical Sciences, University of Naples “Federico II”, Complesso Universitario Monte S. Angelo, Via Cintia 4, 80126 Napoli, Italy; 3 Niogret Ecology Consulting LLC, 13601 Old Cutler Road, Miami, FL 33158, USA

**Keywords:** kairomone, *o*-eugenol, estragole, phenyllactic acid, attractants, invasive species, electroantennography

## Abstract

*Ceratitis capitata*, the Mediterranean fruit fly, is one of the most serious agricultural pests worldwide responsible for significant reduction in fruit and vegetable yields. Eradication is expensive and often not feasible. Current control methods include the application of conventional insecticides, leading to pesticide resistance and unwanted environmental effects. The aim of this study was to identify potential new attractants for incorporation into more environmentally sound management programs for *C. capitata*. In initial binary choice bioassays against control, a series of naturally occurring plant and fungal aromatic compounds and their related analogs were screened, identifying phenyllactic acid (**7**), estragole (**24**), *o*-eugenol (**21**), and 2-allylphenol (**23**) as promising attractants for male *C. capitata.* Subsequent binary choice tests evaluated five semisynthetic derivatives prepared from 2-allylphenol, but none of these were as attractive as 2-allylphenol. In binary choice bioassays with the four most attractive compounds, males were more attracted to *o*-eugenol (**21**) than to estragole (**24**), 2-allylphenol (**23**), or phenyllactic acid (**7**). In addition, electroantennography (EAG) was used to quantify antennal olfactory responses to the individual compounds (**1**–**29**), and the strongest EAG responses were elicited by 1-allyl-4-(trifluoromethyl)benzene (**11**), estragole (**24**), 4-allyltoluene (**14**), *tran*s-anethole (**9**), *o*-eugenol (**21**), and 2-allylphenol (**23**). The compounds evaluated in the current investigation provide insight into chemical structure–function relationships and help direct future efforts in the development of improved attractants for the detection and control of invasive *C. capitata.*

## 1. Introduction

Tropical fruit flies (Diptera: Tephritidae) are among the most economically important pests that threaten global agriculture [1]. Among the exotic fruit flies, the Mediterranean fruit fly (medfly), *Ceratitis capitata* (Wiedemann), is considered one of the most destructive agricultural pests because of its direct damage to many varieties of fruits and vegetables [2,3,4]. The species apparently originated in sub-Saharan Africa; however, increased human mobility and trade in agricultural commodities have increased the incidence of the introduction of exotic fruit flies to the United States [5]. Medfly was first detected in Hawaii in 1910 and in Florida in 1926 and later in 1956, 1962, 1963, 1967, 1981, 1990, 1997, 1998, and 2010 [5,6]. Eradication campaigns in response to detections are extremely laborious and costly [5]. For example, medfly eradication in the Tampa Bay area (Florida) in 1997 cost $US 25 million, and eradication efforts during the past 25 years in California cost nearly $US 500 million [6]. The embargo, loss of market, quarantine regulations, and subsequent job losses further contribute to the overall economic impact of fruit fly incursions in many countries [5]. In March 2015, an outbreak of medfly was reported for the first time in the Dominican Republic and a subsequent ban imposed by the United States resulted in losses of about $US 40 million to Dominican producers [7]. In addition to economic concerns, the high occurrence of pesticide residues in fruits and vegetables and the use of postharvest fumigants like methyl bromide are not desirable by consumers [8,9]. 

Current management strategies for medfly include the use of trimedlure-baited traps for pest detection and monitoring and the release of sterile male flies for population suppression [2,3,4,10,11,12]. Trimedlure, tert-butyl 4(and 5)-chloro-2-methylcyclohexane- l-carboxylate (C_12_H_21_ClO_2_), is a synthetic attractant with a mixture of different diastereomers. Of these isomers, (+)-trimedlure-C was found to be the most attractive [12]. The development of male lures for *C. capitata* has a long history of success. *Angelica archangelica* L. seed oil was used extensively for survey and detection during the eradication program in Florida in 1956 [12,13]. Over a decade later, α-copaene, a natural sesquiterpene hydrocarbon, was identified as the main attractant in angelica seed oil and was reported to be 2–5 times more attractive for *C. capitata* than trimedlure in field tests. While it is highly attractive, α-copaene has limited practical use as a field lure due to its structural complexity and difficulties with synthesis [12]. A study reported by our group also found that the concentration of α-copaene in host plants was correlated with the short-range attraction of male *C. capitata* but not with long-range attraction or olfactory response, as measured by electroantennography (EAG) [14]. Previous research investigated six essential oils—ginger root, orange, manuka, cubeb, angelica seed, and tea tree oil—for their attractancy in laboratory and field tests and EAG response of sterile males and wild *C. capitata*. Results showed that ginger root oil was the most attractive oil in field cage bioassays and elicited the greatest EAG response [15]. These essential oils were further evaluated in short-range bioassays, and tea tree oil, when diluted to 10 µg/µL, showed the highest attraction of sterile male medflies [16]. 

Although considerable progress has been made to identify food-based attractants and host-based kairomones for exotic fruit flies, there is still a need for new and effective attractants to improve the detection and control of invasive medflies. Here, we evaluate a series of aromatic compounds and phenols, a group of chemicals well-known as plant and microbial bioactive metabolites [17,18]. 

**Figure 1:**
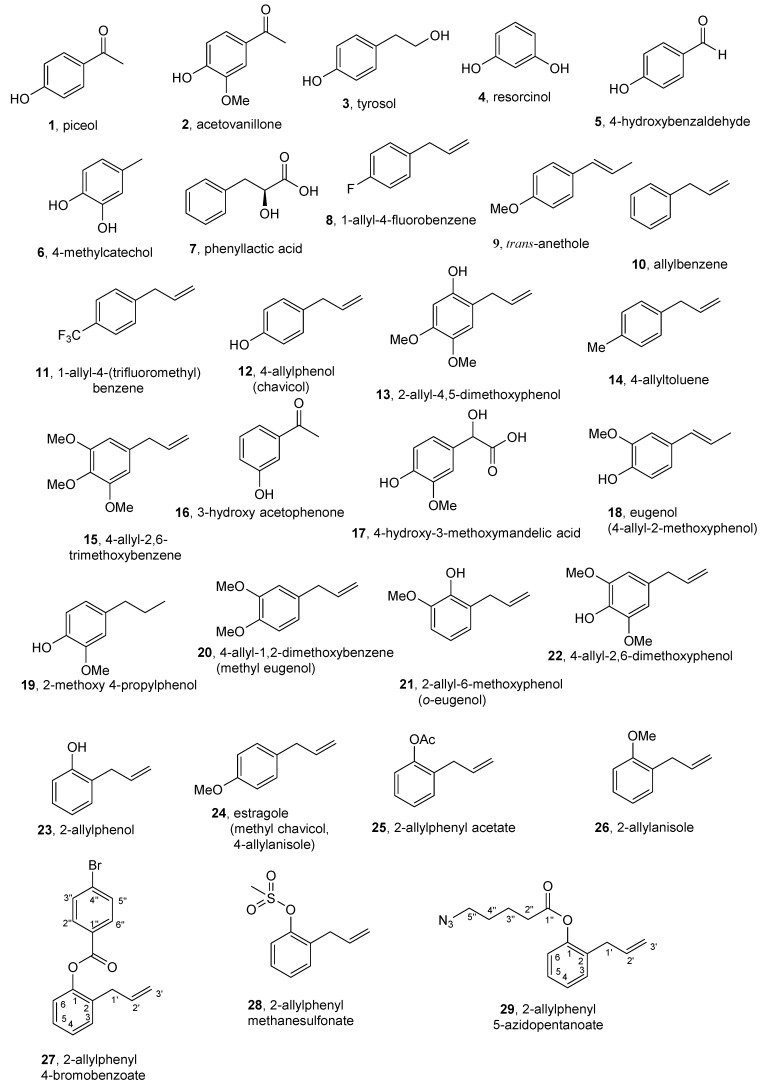
The structures of compounds **1**–**29**.

Our ongoing studies focus on the identification of alkaloids from four native South African Amaryllidaceae species—*Crinum buphanoides* (Welw. ex Baker), *Crinum graminicola* (I. Verd.)*, Cyrtanthus mackenii* (Hook. f.), and *Brunsvigia grandiflora* (Lindl.) [19]—and nine alkaloids belonging to the lycorine subgroup along with the isocrabostyril, tazettine, and crinine subgroups have been isolated [20]. Interestingly, two non-alkaloid compounds, piceol (4-hydroxyacetophenone, **1**, Figure 1) and acetovanillone (apocynin, **2**, Figure 1), were isolated from *C. buphanoides*, while only the former was isolated from *C. graminicola* [21]. In addition, some phytotoxic phenols have been isolated recently from phytopathogenic fungi, including tyrosol (**3**) and resorcinol (**4**) (Figure 1), purified from the culture filtrates of *Dothiorella vidmadera* (DAR78993), a pathogen involved in the Botryosphaeria dieback of grapevine [22] and 4-hydroxybenzaldehyde (**5**, Figure 1) purified from the solid cheatgrass (*Bromus tectorum* L.) culture of a *Fusarium* strain belonging to the *F. tricinctum* (Corda) species complex [23]. 

The findings above prompted the current collaboration to test these compounds for semiochemical activity using a laboratory strain of *C. capitata*. Specifically, we (i) investigated short-range attraction of male *C. capitata* to 29 structurally related natural and synthetic aromatic compounds (Figure 1) using small cage laboratory bioassays, (ii) conducted EAG analyses to quantify olfactory chemoreception of these compounds, and (iii) discuss the structure–activity relationships of these compounds to facilitate identification of promising candidate attractants for future research. In addition, we describe the synthesis of five derivatives of 2-allylphenol (**23**) used in this study. 

## 2. Results and Discussion

Aromatic compounds with different functional groups isolated from plants (**1** and **2**), fungi (**3**–**5**), and their commercially available analogs (**6**–**22**, **24**) and some semisynthetic derivatives (**23**, **25**–**29**) were investigated for potential attraction of male *C. capitata*. Five derivatives (**25**–**29**) (Figure 1) were semisynthesized from the commercially available 2-allylphenol (**23**, one of the active attractants of *C. capitata* in the initial bioassay; see the Materials and Methods section). 

In particular, 2-allylphenol (**23**), by reaction with Ac_2_O and pyridine, was converted into the corresponding 1-*O*-acetylderivative (**25**). Its ^1^H-NMR spectrum differed from that of **23** for the presence of the typical singlet of the acetyl group at δ 2.32 and was very similar to that previously reported Gresser et al. [24]. Further confirmation was given by an ESI-MS spectrum, which showed the protonated form [M + H]^+^ at *m/z* 177.

By reaction with an ethereal solution of diazomethane, **23** was converted into the corresponding methyl ether (allylanisole, **26**). Its ^1^H NMR spectrum differed from that of **23** for the significant presence of the singlet of methoxy group at δ 3.83 and was very similar to that previously reported [25]. Furthermore, its ESI-MS showed the protonated form [M + H]^+^ at *m/z* 149.

2-Allylphenol (**23**), by reaction with 4-bromobenzoic acid, yielded its corresponding *p*-bromobenzoyl ester (**27**). Its ^1^H-NMR spectrum, compared with that of **23**, showed the typical signals pattern of the aromatic *para*-disubstituted protons, appearing as two doublets at δ 8.08 and 7.69 (*J* = 8.7 Hz). In addition, the ESI-MS spectrum showed the protonated form [M + H]^+^ of typical signals due to the presence of ^79^Br and ^81^Br isotopes peaks at *m/z* 317 [M + H]^+^ and 319 [M + 2 + H]^+^, respectively.

2-Allylphenol (**23**), by reaction with mesyl chloride in pyridine, afforded the corresponding mesyl ester (**28**). Its ^1^H-NMR spectrum was differed from **23** by the significant presence of the singlet of the methyl group at δ 3.20 and was very similar to that previously reported by Lei et al. [26]. Its ESI-MS spectrum showed the protonated form [M + H]^+^ at *m/z* 213.

2-Allylphenol (**23**), by reaction with 5-azidopentanoic acid, afforded the 5-azido pentanoyl derivative (**29**). Its ^1^H-NMR spectrum differed from that of allylphenol for the presence of the signals of 5-azidopentanoyl residue, appearing at 3.32 (t, *J* = 6.8 Hz, 2H, H-5″), 3.21 (d, *J* = 6.6 Hz, 2H, H-1′), 2.64 (t, *J* = 7.0 Hz, 2H, H-2″), 1.91–1.84 (m, 2H, H-3″), and 1.79−1.74 (m, 2H, H-4″). Its ESI-MS spectrum showed the protonated form [M + H]^+^ at *m/z* 260.

All the natural, commercially available and synthetic compounds were assayed against *C. capitata* sterile males using short-range attraction bioassays and EAG analyses, as detailed in the Material and Methods section.

Two short-range bioassays were carried out. In Experiment 1, paired *t*-tests found that male response to compounds **7**, **9**, **11**, **13**, **21**, **23**, **24**, and **27** was higher than the response to the associated solvent control (Table 1). Response of flies was converted to Attraction Index for comparisons among these eight compounds**.** There was an effect of type of chemical on Attraction Index. (*F*_7,32_ = 16.69, *p* < 0.0001). The highest index was found with chemical **7** (0.63 ± 0.26), intermediate indexes with chemicals **24**, **21**, and **23** (0.41 ± 0.19, 0.37 ± 0.11, and 0.28 ± 0.08, respectively), and the lowest indexes with chemicals **9**, **11**, **13**, and **27** (ranging from 0.02 ± 0.01 to 0.08 ± 0.06).

These results demonstrated the importance of the functional groups and their position on the benzene ring. In particular, the side chain of **7** was the most important moiety to stimulate attraction of males in Experiment 1. The presence of the allyl group was also a critical factor, as evidenced by little or no attraction observed with compounds lacking this moiety, as in **1**–**6**, **16**, and **17**. The same result was obtained when the allylic chain was modified by isomerization of the double bond, as in **9** and **18**, or by the presence of a propyl residue, as in **19**. However, other substitutions on the aromatic ring of allylbenzene (**10**) were needed to confer activity, as **10** elicited no response. When the number of substituents on the aromatic ring increased, those with three functional groups lost activity, as in **13**, **15**, and **22**. Among compounds having two substituents, as in **20** and **21**, only the latter, having a substituent *ortho*-located with respect to the allyl group, was active. Although methyl eugenol (**20**) is a male attractant for *Bactrocera* *dorsalis* (Diptera: Tephritidae) [27], male medflies showed poor response to compound **20**. Among the *ortho*- and *para*-monosubstituted allyl benzenes, only **23** and **24** displayed activity while the others (**8**, **11**, **12**, and **14**) were inactive. This suggests that the efficacy was dependent upon the substituents and their volatility and fragrance. Furthermore, comparing the activity of the four esters (**25**, **27**–**29**) and one ether of **23** with that of the parent compound, they were all less attractive. Thus, the presence of a free hydroxyl and the *ortho*-allyl group was important for the activity. It should also be noted that some of the compounds tested could be potential repellents; however, that determination would require bioassays with a different experimental design (e.g., a known attractant vs. a combination of attractant plus potential repellent). Further investigations are needed to assess potential repellent properties of compounds deemed non-attractive in this study.

Lipophilicity, expressed as the logarithm of the octanol–water partition coefficient (log *P*), is often correlated with biological activity. Log *P* values of a series of test chemicals often follow predictable trends in biological assays [28]. Compounds with lower log *P* values are classified as polar, while those with higher log *P* values are considered more lipophilic with better membrane permeability [29]. However, we did not observe any correlation between Log *P* values for compounds **1**–**28 [30]** and **29** [31] and male response in Experiment 1 (*r* = 0.03037, n = 29, *p* = 0.8757) (Table 1).

When pairwise comparisons of the four most attractive compounds from Experiment 1 were tested in Experiment 2, there were clear choices among most chemicals (Table 2). More males were attracted to compound **21,** and fewer males were attracted to compound **7** in all bioassays. Attraction to compounds **23** and **24** was intermediate, with no difference in attraction when **23** and **24** were tested together. Phenyllactic acid (**7**) has been isolated from cultures of *Lactobacillus plantarum* [32] and may function as a food-based attractant. Estragole (**24**) is more likely a kairomone, since it occurs in a variety of essential oil-bearing plants such as basil, tarragon, chervil, fennel, clary sage, anise, and rosemary [33], as well as in the leaves of various avocado cultivars [34], ripe apple [35] and citrus fruits [36].

These results support that both the presence of the allyl residue, and substituents on the aromatic ring are key structural features that confer attraction of male *C. capitata*.

**Figure 2:**
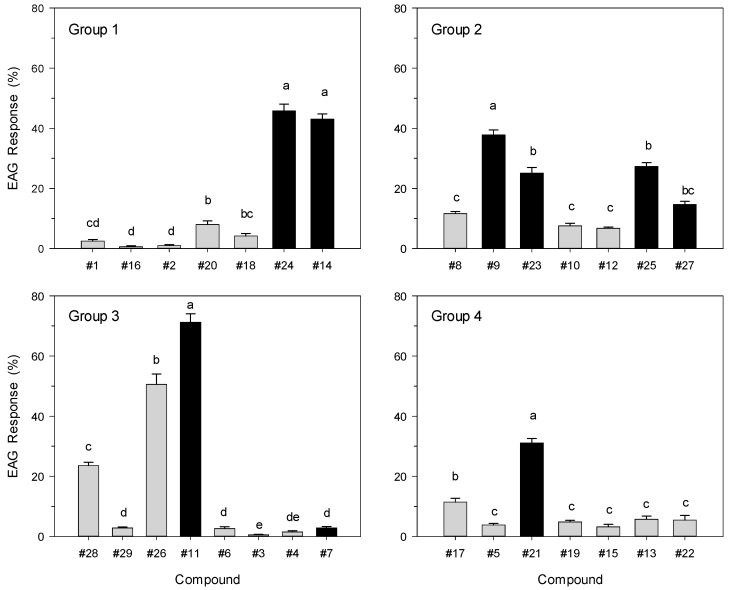
Mean (± SE) electroantennogram (EAG) responses of male *C. capitata* to 1 mL doses of saturated vapor from compounds **1**–**29** (neat material). Analyses were conducted using four groupings of randomly chosen samples; responses were measured from 10 replicate males per group. All EAG responses expressed as normalized percentages relative to the standard reference sample (tea tree essential oil, 1 mL saturated vapor). Black bars indicate compounds found attractive in short-range bioassays; gray bars indicate non-attractive compounds. Within each group comparison, bars topped with the same letter are not significantly different (Tukey mean separation, *p* < 0.05).

In EAG analyses (Figure 2), there were significant differences in mean olfactory response to test chemicals observed in all four groupings (Group 1: *F*_6, 68_ = 185.02, *p* < 0.001; Group 2: *F*_6, 68_ = 95.97, *p* < 0.001; Group 3: *F*_7, 76_ = 375.321, *p* < 0.001; and Group 4: *F*_6, 68_ = 78.33, *p* < 0.001). In general, strong EAG responses were elicited by compounds that were observed to be attractive in short range bioassays (indicated by black bars in Figure 2). Of the nine highest-ranked chemicals in Experiment 1 (Table 1), only compound **7** had a low amplitude depolarization peak (Figure 2, Group 3). This may have been due to differences in sample preparation between the bioassays and EAG analyses. The former used 10% dilutions in acetone, whereas the latter used chemicals in their neat form. With compound **7**, the neat material at 24 °C was in solid state, and the dry crystals may not have generated significant volatiles in the headspace of the EAG sample bottle. It is also possible that compound **7**, when presented in bioassays, was detected by contact chemoreceptors on the tarsi rather than by antennal olfactory receptors.

In addition, there were two chemicals that elicited higher than expected EAG responses. Of the twenty low-ranked (i.e., non-attractive) chemicals from the behavioral assays (indicated by gray bars in Figure 2), all displayed weak EAG responses except for compounds **26** and **28**, which produced relatively high amplitude EAG peaks (Figure 2, Group 3). Potential explanations for this observation are that these two chemicals may be (i) true attractants, but the insects are not at the proper physiological stage to respond appropriately (all test insects were sterile virgin males of the same age); (ii) synergistic attractants, not behaviorally active alone but increasing response when combined with primary attractants (e.g., putrescine, a synergist when combined with ammonia as a tephritid food-based attractant [37]; (iii) repellents, which would also be detected by antennal olfactory receptors (e.g., ammonia, a protein feeding cue attractive at low doses but repellent at high dose) [38]; or (iv) other biologically relevant compounds in the environment but unrelated to attraction behavior.

## 3. Materials and Methods 

### 3.1. General Experimental Procedures 

^1^H NMR spectra were recorded at 500 and 400 MHz in CDCl_3_ on Varian (Varian, Palo Alto, CA, USA) and Bruker (Bruker, Karlsruhe, Germany) spectrometers. The same solvent was used as internal standard. Liquid chromatography-mass spectrometry (LC-MS) analysis was performed on an Agilent HPLC 1100 VL instrument (Agilent Technologies, Milan, Italy) equipped with an electrospray ionization source (positive ion mode, ESI+). An Eclipse XBD-C18 column (150 mm × 4.60 mm, 5 μm) was used, adopting 0.1% formic acid: MeOH (35:65) as the eluant (flow rate: 0.4 mL/min). Analytical and preparative TLC were performed on silica gel (Kieselgel 60, F_254_, 0.25 and 0.5 mm respectively) plates. The spots were visualized by exposure to UV radiation (253 nm) or iodine vapour or by spraying first with 10% H_2_SO_4_ in MeOH and then with 5% phosphomolybdic acid in EtOH, followed by heating at 110 °C for 10 min. Column chromatography was performed using silica gel (Merck, Kieselgel 60, 0.06–0.200 mm, KgaA, Darmstadt, Germany). Log *P* values were computed by using ChemDraw 18.0 Ultra (**1**–**28**) [30] and ChemAxon 19.10 (**29**) [31].

### 3.2. Natural and Synthetic Compounds

The compounds used for the study (Figure 1) are natural and synthetic aromatic derivatives with different functional groups. In particular, piceol (**1**) and acetovanillone (**2**) were purified from the organic extract of *Crinum buphanoides* bulbs, a native South African Amaryllidaceae plant [21]. Tyrosol (**3**) and resorcinol (**4**) were isolated from the culture filtrate of the fungus *Dothiorella vidmadera*, a pathogen involved in the Botryosphaeria dieback of grapevine [22]. 4-Hydroxybenzaldehyde (**5**) was isolated from the solid cheatgrass (*Bromus tectorum*) culture of a *Fusarium* strain belonging to the *F. tricinctum* species complex [23]. Compound **12** (Cas# 501-92-8) was purchased from Parkway Scientific (New York, NY, USA), **13** (Cas# 59893-87-7) was purchased from Enamine Ltd. (Monmouth Junction, NJ, USA), and **15** (Cas# 487-11-6) was purchased from BOC Sciences (Shirley, NY, USA). All other compounds (**6** (Cas# 452-86-8), **7** (Cas# 20312-36-1), **8** (Cas# 1737-16-2), **9** (Cas# 4180-23-8), **10** (Cas# 300-57-2), **11** (Cas# 1813-97-4), **14** (Cas# 3333-13-9), **16** (Cas# 121-71-1), **17** (Cas# 55-10-7), **18** (Cas# 97-53-0), **19** (Cas# 2785-87-7), **20** (Cas# 93-15-2), **21** (Cas# 579-60-2), **22** (Cas# 6627-88-9), **23** (Cas# 1745-81-9), and **24** (Cas# 140-64-0) were supplied from Sigma-Aldrich Ltd. (St. Louis, MO, USA).

#### Synthesis of 2-Allylphenol Derivatives **25**–**29**

*2-allylphenil acetate (**25**).* 2-allylphenol (**23**, 120 µL), dissolved in pyridine (50 µL), was converted in its corresponding acetyl ester (**25**) by acetylation with Ac_2_O (50 µL). The reaction was carried out under stirring for 12 hours at room temperature. It was stopped with MeOH, and the azeotrope formed by addition of C_6_H_6_ was evaporated under N_2_ stream. The residue (125.2 g) was then purified by column chromatography (CC) on silica gel eluted with *n*-hexane:EtOAc (9:1) yielding **25** (119.3 mg) as an amorphous oil. Its ^1^H NMR data were very similar to those previously reported by Gresser et al. [24]; ESI+/MS: *m*/*z* 177 [M + H]^+^.

*2-allylanisole (**26**).* An ethereal solution of CH_2_N_2_ was added to a solution of 2-allylphenol (**23**, 120 µL) in MeOH (120 µL) to obtain a persistent yellow color. The reaction was carried out at room temperature under stirring and was stopped after 24 h by evaporation under an N_2_ stream. The crude residue (130.1 mg) was purified by CC, using *n*-hexane:EtOAc (9:1) as eluent, to give 100 mg of 2-allylanisole (**23**) as a homogeneous compound. Its ^1^H NMR data were very similar to those already reported in literature [25]; ESI+/MS: *m/z* 149 [M + H]^+^.

*2-allylphenyl-4-bromobenzoate (**27**).* To 2-allylphenol (**23**, 180 µL), dissolved in anhydrous MeCN (600 µL), DMAP (120.0 mg) and *p*-bromobenzoilchloride (120.0 mg) were added. The reaction mixture was left under stirring for 24 h. It was then quenched with a 1 N NaHCO_3,_ extracted with EtOAc and washed with H_2_O. The residue obtained by evaporation (244.6 mg) was then purified by CC eluted with *n*-hexane:acetone (9:1), affording the *p*-bromobenzoyl ester of 2-allylphenol (**27**, 102.2 mg). Derivative **27** had ^1^H NMR (400 MHz, CDCl_3_, δ, ppm); δ 8.08 (d, *J* = 8.7 Hz, 2H, H-2″ and H-6″), 7.69 (d, *J* = 8.7 Hz, 2H, H-3″ and H-5″), 7.32 (m, 2H, H-5 and H-3), 7.26 (td, *J* = 7.3 and 2.5 Hz, 1H, H-4), 7.18 (br d, *J* = 7.3 Hz, 1H, H-6), 5.93 (ddt, *J* = 17.1, 10.6 and 6.6 Hz, 1H, H-2′), 5.05 (dd, *J* = 10.6 and 2.9 Hz, 1H, H-3′A), 5.02 (dd, *J* = 17.1 and 2.9 Hz, 1H, H-3′B), and 3.21 (d, *J* = 6.6 Hz, 2H, H_2_-1′); and ESI+/MS: *m*/*z* 319 [M + 2 + H]^+^, 317 [M + H]^+^.

*Mesyl ester of 2-allylphenol (**28**).* Two hundred and fifty µL of mesyl chloride were added to a solution of 2-allylphenol (**23**, 60 µL) in CH_2_Cl_2_ (300 µL) together with 60 µL of pyridine. The reaction mixture was kept overnight and then quenched with a 1 N solution of HCO_3_^−^. The mixture was then extracted with EtOAc, and the resulting organic extract (63.1 mg) was purified by preparative TLC eluted with CHCl_3_, affording the methyl ester derivative of 2-allylphenol (**28**, 52.6 mg). ^1^H NMR data of **28** were very similar to those previously reported by Lei et al. [26]; ESI+/MS: *m/z* 213 [M + H]^+^.

*Azidopentanoyl ester of 2-allylphenol (**29**).* 2-allylphenol (**23**, 120.0 µL) dissolved in anhydrous CH_2_Cl_2_ (600 µL) and pyridine (120 μL), and DCC (*N*,*N*-dicyclohexylcarbodiimide) (40 mg) and 5-azidopentanoic acid (120 μL) were added. The reaction was left at room temperature for 4 days. The reaction was stopped by evaporation under N_2_. The residual oil (178.2 mg) was then purified by CC eluted with *n*-hexane:acetone (9:1), affording 5-azidopentanoyl ester of 2-allylphenol (**29**, 43.8 mg). Derivative **29** had ^1^H NMR (400 MHz, CDCl_3_, δ, ppm); δ 7.32 (m, 2H, H-5 and H-3), 7.26 (td, *J* = 7.3 and 2.5 Hz, 1H, H-4), 7.18 (br d, *J* = 7.3 Hz, 1H, H-6), 5.92 (ddt, *J* = 17.1, 10.6 and 6.6 Hz, 1H, H-2′), 5.05 (dd, *J* = 10.6 and 2.9 Hz, 1H, H-3′A), 5.02 (dd, *J* = 17.1 and 2.9 Hz, 1H, H-3′B), 3.32 (t, *J* = 6.8 Hz, 2H, H-5″), 3.21 (d, *J* = 6.6 Hz, 2H, H-1′), 2.64 (t, *J* = 7.0 Hz, 2H, H-2″), 1.91–1.84 (m, 2H, H-3″), and 1.79–1.74 (m, 2H, H-4″).; ESI+/MS: *m/z* 260 [M + H]^+^.

### 3.3. Insects

Sterile male *C. capitata* were obtained from the Programa Moscamed mass rearing facility (El Pino, Guatemala), where they were irradiated as pupae 2 d prior to emergence with 95 Gy of gamma radiation from a Co60 source. These are the temperature-sensitive lethal strain flies [39] that are used for the preventative release program [40] in Florida. Thus, only males were obtained, and only virgin males were used for testing. Irradiated pupae were shipped initially to the USDA-APHIS Medfly Project (Sarasota, FL, USA) and then to the USDA-ARS Subtropical Horticulture Research Station in Miami, FL. Holding conditions at Miami consisted of a 12/12 h L/D photoperiod, 25 ± 2 °C, and 75 ± 5% RH. Pupae were placed in collapsible cages (30.5 × 30.5 × 30.5 cm). After eclosion, adult flies were provided with water (2% agar blocks) and food (3:1 mixture of cane sugar and yeast hydrolysate). Flies used for all studies were 5 to 10 d-old, sexually mature sterile virgin males. Only sterile flies were available for use in this research because there are no wild populations in Florida. Previous research, however, has found that response of sterile males to semiochemicals is similar to response of wild males (e.g., Reference [15]).

### 3.4. Short-Range Bioassays

Small cage bioassays were used to quantify the short-range attraction of sterile male *C. capitata* using a modified version of the binary choice tests [41]. All observations were carried out at room temperature as described above in small collapsible cages (20.3 × 20.3 × 20.3 cm) into which 50 flies were introduced 1 h prior to the start of each experiment. Tests were initiated by introducing two Petri dishes (53 mm diameter and 12 mm height) with substrates positioned symmetrically (37 mm apart). After 30 min, the number of flies at each dish was recorded. Experiment 1 compared the response to each individual chemical (10 µL of a 10% dilution in acetone) with the response to a paired solvent control (10 µL acetone). Test substrate or control was added to the center of a filter paper disk (Whatman #1, 3.5 cm diam). The filter paper disk was air-dried briefly to allow the solvent to evaporate and was placed into the middle of a Petri dish. Bioassays were replicated five times, and the position of substrates reversed between replicates. Flies and Petri dishes were used only once, and cages were washed with acetone between experiments to eliminate potential residual chemicals.

Pairwise comparisons of the chemicals that elicited the highest response in the initial tests were then conducted in Experiment 2 (compounds **7**, **21**, **23**, and **24**). The response to each individual chemical (10 µL of a 10% dilution in acetone) was compared with the response to each other selected chemicals in this two-choice test bioassay with all possible combinations tested (**7** vs. **21**, **7** vs. **23**, **7** vs. **24**, **21** vs. **23**, **21** vs. **24**, **23** vs. **24**). There were ten replicates of the pairwise comparisons, with each pair tested in separate cages at the same time.

### 3.5. Electroantennography (EAG) Analysis

Peripheral olfactory responses were recorded from antennae of male *C. capitata* using a Syntech EAG system (Syntech Original Research Instruments, Hilversum, Netherlands) and methods developed by Kendra et al. [37,38,42]. Test substrates consisted of the 29 compounds, each 20 mg neat material. The standard reference sample (positive control) was tea tree oil, 20 mg (Essential Oil India-SAT Group, Kannauj, India), shown previously to elicit strong EAG responses in male medflies [15]. Each substrate was placed into a separate 250 mL hermetic glass bottle equipped with a lid containing a short thru-hull port (Swagelok, Solon, OH, USA) and silicone septum (Alltech, Deerfield, IL, USA). Sample bottles were sealed and equilibrated overnight at 24°C to allow for headspace saturation with volatiles.

Freshly dissected antennal preparations (whole head mounts) were secured between electrodes using salt-free conductive gel (Spectra 360, Parker Laboratories, Fairfield, NJ, USA) and placed under a stream of humidified air, purified with activated charcoal granules, at a flow rate of 400 mL/min. Using gas-tight syringes (SGE Analytical Science, Victoria, Australia), samples of saturated vapor were withdrawn from the test bottles, injected into the airstream, and presented to the antennae. In each recording session, samples (fixed 1 mL doses) were delivered in the following order: the tea tree standard, test chemicals in random order, a clean air injection (negative control), and a final standard injection. There was a 2-min interval (clean air flush) between injections to prevent antennal adaptation (diminished EAG response resulting from repeated exposure to chemical stimuli). Due to the large number of test chemicals, EAG analyses were conducted using four groupings; each group compared olfactory responses to seven or eight chemicals, randomly chosen, and responses were measured from ten replicate females.

EAG responses to test substrates were measured initially in millivolts (peak height of depolarization) and then normalized to percentages relative to the EAG response obtained with the reference sample. Normalization corrects for time-dependent variability (gradual decline) in antennal performance and allows for comparison of relative EAG responses obtained with different substrates [37,43,44,45] and with different cohorts of insects [37,38]. Finally, any response recorded with the negative control was subtracted from the normalized test responses to correct for “pressure shock” caused by injection volume. All statistical analyses were performed using the corrected normalized EAG values.

### 3.6. Statistical Analysis

Pair *t*-tests were used to test for differences in number of males attracted to each choice in the binary choice tests in Experiments 1 and 2 (Proc TTEST; SAS Institute, 2016) [46]. Male response was converted to Attraction Index (number attracted to the compound minus number attracted to the control divided by total number of males tested) [47] to compare the eight compounds that attracted more males than the paired control in Experiment 1. One-way ANOVA and Tukey’s mean separation tests were used to determine effect of chemical on Attraction Index in Experiment 1 and on olfactory responses in EAG analyses. When necessary, data were transformed prior to ANOVA to satisfy conditions of equal variance [48]; non-transformed means ± standard deviations are presented.

## 4. Conclusions

In an effort to find effective new attractants for *C. capitata*, we investigated 29 structurally related aromatic compounds in short range bioassays and EAG analyses. The combined results identified phenyllactic acid (**7**), estragole (**24**), *o*-eugenol (**21**), and 2-allylphenol (**23**) as promising candidates for sexually mature males. Of these four compounds, *o*-eugenol (**21**) was observed to be the most attractive in binary choice tests. The presence of the allyl residue and substituents on the aromatic ring appear to be key structural features that confer attraction to these compounds. This study provides insight into the attractiveness of structural variants of aromatic compounds with various substituent groups to male *C. capitata*. Another promising approach could be the synthesis of estragole analogs with allyl groups at different sites on the aromatic ring. In addition, further studies are needed to evaluate these compounds, alone and in combination, to determine their efficacy in the field.

## Figures and Tables

**Table 1:** Log *P* values and number (mean ± std dev) of sterile male *C. capitata* attracted to compounds **1**–**29** presented in binary choice bioassays against control (Experiment 1).

Compound	Log *P* Values ^£^	Number Responding
phenyllactic acid (**7**)	1.16	32.6 ± 12.4 *
estragole (methyl chavicol, 4-allylanisole) (**24**)	2.96	21.0 ± 9.0 *
2-allyl-6-methoxyphenol (*o*-eugenol) (**21**)	2.57	20.2 ± 4.3 *
2-allylphenol (**23**)	2.7	16.0 ± 3.8 *
2-allylphenyl acetate (**25**)	2.67	10.0 ± 8.7
4-allyltoluene (**14**)	3.57	8.8 ± 8.3
*tran*s-anethole (**9**)	2.91	6.0 ± 2.3 *
2-allylphenyl 4-bromobenzoate (**27**)	5.4	5.0 ± 2.3 *
1-allyl-4-(trifluoromethyl)benzene (**11**)	4.01	3.2 ± 1.3 *
2-methoxy 4-propylphenol (**19**)	2.84	3.0 ± 3.3
2-allylphenyl methanesulfonate (**28**)	1.99	3.0 ± 0.7
4-hydroxybenzaldehyde (**5**)	1.39	2.8 ± 2.6
2-allylphenyl 5-azidopentanoate (**29**)	3.65	2.6 ± 1.3
1-allylbenzene (**8**)	3.24	2.2 ± 1.1
4-allylphenol (chavicol) (**12**)	2.7	2.2 ± 1.1
tyrosol (**3**)	1.35	2.0 ± 1.6
resorcinol (**4**)	1.26	2.0 ± 2.5
4-methylcatechol (**6**)	1.74	1.6 ± 2.1
2-allyl-4,5-dimethoxyphenol (**13**)	2.44	1.6 ± 0.5 *
allylbenzene (**10**)	3.09	1.4 ± 0.9
4-allyl-1,2-dimethoxybenzene (= methyl eugenol) (**20**)	2.83	1.4 ± 0.5
3-hydroxy acetophenone (**16**)	0.96	1.2 ± 1.3
4-hydroxy-3-methoxymandelic acid (**17**)	0.36	1.2 ± 1.3
2-allylanisole (**26**)	2.96	1.2 ± 0.8
4-allyl-2,6-trimethoxybenzene (**15**)	2.71	1.0 ± 0.7
eugenol (4-allyl-2-methoxyphenol) (**18**)	2.52	0.8 ± 0.8
piceol (**1**)	0.96	0.6 ± 0.9
4-allyl-2,6-dimethoxyphenol (**22**)	2.44	0.6 ± 0.9
acetovanillone (**2**)	0.83	0.4 ± 0.5

*Number of flies (*n *= 5 replicates, 50 flies per replicate) on treated paper was greater than number on solvent control (paired *t*-test, *p* < 0.05). **^£ ^**as detailed in the Material and Methods section.

**Table 2:** Number (mean ± std dev) of male *C. capitata* attracted to each choice in pairwise comparisons of the top four compounds in Experiment 2 for 10 replicate tests with 50 flies per replicate.

Compounds Tested	Number Responding to Each Compound in Bioassay						
	7	21	23	24	*t*	df	*p*
**7** versus **21**	2.1 ± 2.3	17.3 ± 4.4			10.75	16.6	<0.0001
**7** versus **23**	3.2 ± 5.7		14.2 ± 4.3		5.3	22	<0.0001
**7** versus **24**	2.4 ± 2.0			16.1 ± 5.6	7.97	13.7	< 0.0001
**21** versus **23**		17.8 ± 6.0	7.8 ± 2.8		4.54	15.6	0.0001
**21** versus **24**		17.1 ± 5.7		7.8 ± 5.4	4.14	22	0.0004
**23** versus **24**			10.3 ± 3.2	10.4 ± 4.9	0.05	22	0.9809
